# Supplementary material for: Inhibitory columnar feedback neurons are involved in motion processing in Drosophila
Source: eLife. 2026 May 26;14:RP108529. doi: 10.7554/eLife.108529 (PMC13211874; doi:10.7554/eLife.108529)
Supplement: Supplementary file 1. — Neurons were identified based on either colocalization of the InSITE expression pattern with a GABA antibody, followed by single cell Flp-Out experiments, or a InSITE-Gal4-Gad1-intersection strategy. [file elife-108529-supp1.docx]

Supplementary table 1

| InSITE  OFF-motion deficit | GABA colocalization/Flp-Out  GABA pos – GABA neg | | Gad1-intersection | Figure |
| --- | --- | --- | --- | --- |
| PBac{IT.GAL4}0787 | C2 | Tm9 | C2, (CT1) | Figure 1a-c, Figure 1-figure supplement 1d |
| PBac{IT.GAL4}0564 | C2 |  | C2, (C3,CT1) | Figure1a-c, Figure 1-figure supplement 1d |
| PBac{IT.GAL4}0301 | C2 |  | C2, Mi | Figure 1a-c, Figure 1-figure supplement 1d |
| PBac{IT.GAL4}0940 | C2 |  | C2,(C3,CT1),Mi | Figure 1a-c, Figure 1-figure supplement 1d |
| PBac{IT.GAL4}0470 |  | T1, TM4 | (C3, CT1) | (not shown) |
| PBac{IT.GAL4}0396 | C2 (no Flp-Out clone found) | T1, L4, Tm2 |  | (not shown) |
| PBac{IT.GAL4}0096 |  | Tm9 | - | (not shown) |

| InSITE  ON-motion deficit | GABA colocalization/Flp-Out  GABA pos – GABA neg | | Gad1-intersection |  |
| --- | --- | --- | --- | --- |
| PBac{IT.GAL4}0619 | - | - | C2, (C3, CT1) | Figure 1d |
| PBac{IT.GAL4}0913 | - | - | C2, (C3, CT1) | Figure 1d |
| PBac{IT.GAL4}0669 | - | - | C2, (C3, CT1) | Figure 1d |
| PBac{IT.GAL4}0974 | - | - | (C3, CT1), LT | Figure 1-figure supplement 1f |
| PBac{IT.GAL4}1037 | - | - | Mi1^new^, (C3, CT1) | Figure 1-figure supplement 1f |
| PBac{IT.GAL4}0756 | - | - | - | Figure 1-figure supplement 1f |
| PBac{IT.GAL4}0518 | - | - | (C3, CT1) | Figure 1-figure supplement 1f |
| PBac{IT.GAL4}0980 | - | - | Mi1^new^, (C3, CT1) | Figure 1-figure supplement 1f |
| PBac{IT.GAL4}0081 | - | - | (C3), LT | Figure 1-figure supplement 1f |
| PBac{IT.GAL4}0168 | - | - | (C3), Mi, LT | Figure 1-figure supplement 1f |
| PBac{IT.GAL4}0651 | - | - | (C3), LT | Figure 1-figure supplement 1f |
